# Nursing Intervention Countermeasures of Robot-Assisted Laparoscopic Urological Surgery Complications

**DOI:** 10.1155/2021/8223941

**Published:** 2021-11-30

**Authors:** Xushu An, Jinyuan Zhou, Xuenan Ma, Bingbing Song

**Affiliations:** Cancer Hospital Affiliated to Harbin Medical University, Heilongjiang, Harbin 150000, China

## Abstract

The objective is to explore the application effect of comprehensive nursing intervention in prevention of lower extremity deep vein thrombosis and pulmonary embolism in urological patients undergoing laparoscopic and robot-assisted laparoscopic surgery. From April 2019 to April 2020, 200 patients who received urological laparoscopic surgery and robot-assisted laparoscopic surgery were selected. According to the random number table method, they were divided into control group and observation group, 100 cases in control group and 100 cases in observation group. Patients in control group received routine nursing, while patients in observation group received comprehensive nursing intervention. The skin condition, swelling, pain, and occurrence of deep venous thrombosis and pulmonary embolism of lower extremities in 2 groups were observed. The experimental results showed that the lower limb swelling, lower limb pain, and lower limb deep vein thrombosis in the control group were significantly higher than those in the observation group, but all patients were cured and discharged after taking effective symptomatic treatment and nursing measures in time. In the control group, pulmonary embolism occurred in 3 patients, all of whom died. There was no significant difference in skin changes of lower limbs (*P* > 0.05), and there were significant differences in other skin changes (*P* < 0.05). It proved that comprehensive nursing intervention can effectively prevent the formation of lower extremity deep vein thrombosis and pulmonary embolism in urological patients undergoing laparoscopic and robot-assisted laparoscopic surgery with high-risk factors.

## 1. Introduction

In recent years, with the development of science and technology, minimally invasive surgery has become one of the mainstream directions in the development of urology, in which robotic surgery, as a new minimally invasive surgical method, is gradually being applied in the field of surgery [1]. The da Vinci surgical system (DVSS) is widely used in clinical practice. It has a wide scope and mature technology and is gradually applied in urology including nephrectomy, renal tumor enucleation, adrenal tumor resection, pyeloplasty, total cystectomy, and radical prostatectomy [2]. Robot-assisted laparoscopic radical prostatectomy is the minimally invasive surgery with the most obvious advantages compared with open and traditional laparoscopic surgery among all urological robotic surgeries at present [3]. Traditional open surgery requires a large incision in the lower abdomen, resulting in large surgical trauma and large blood loss [4]. However, the development of laparoscopic radical prostatectomy is relatively slow, with no obvious advantages compared with open surgery, and its clinical application is limited. The advent of the da Vinci robot-assisted system has made a qualitative leap in laparoscopic surgery for prostate cancer, and its operation is stable and dexterous, showing obvious advantages [5]. At present, robot-assisted laparoscopic radical prostatectomy has accounted for about 80% of radical prostatectomy in European and American countries with high incidence of prostate cancer [6]. Compared with open and traditional laparoscopic surgery, robotic surgery has the advantages of less bleeding, fewer complications, faster postoperative recovery, and shorter hospital stay and can achieve the same or better oncology and functional results [7] although the operation time is longer and the cost is higher.

Postoperative complications mainly include rectal injury, bleeding, urinary leakage, urinary incontinence, and erectile dysfunction. These complications if not early detected and timely treated can lead to serious consequences, so it is necessary to understand the complications and targeted prevention measures and reduce the incidence of complications, so as to improve the success rate of surgery [8]. Therefore, international research continues. Yaxley et al. found that children with recurrent obstruction after pyeloplasty usually need reoperation. We previously reported 16 cases of failed pyeloplasty in children with robot-assisted laparoscopic repair and concluded that short- and medium-term outcomes were comparable to open resurgical repair [9]. Davis reported on the first series of robot-assisted urinal residual resection for children. Materials and methods: we reviewed the medical records of all children who underwent robot-assisted ural residual resection between 2010 and 2016 [10]. Escolino et al. proposed a new minimally invasive surgical technique for primary obstructive giant ureter (POM), robot-assisted laparoscopic extravesical separation trans-deltoid replantation (RADECUR), and reported a group of 13 patients [11]. On the basis of current research, this paper proposed to explore the application effect of comprehensive nursing intervention in the prevention of urological laparoscopic and robot-assisted laparoscopic surgery in patients with high risk of lower extremity deep vein thrombosis and pulmonary embolism. It proved that comprehensive nursing intervention can effectively prevent the formation of lower extremity deep vein thrombosis and pulmonary embolism in urological patients undergoing laparoscopic and robot-assisted laparoscopic surgery with high-risk factors.

## 2. Data and Methods

### 2.1. Experimental Subjects

A total of 200 patients who received laparoscopic or robot-assisted laparoscopic surgery in the department of urology of the first affiliated hospital of a university from April 2019 to April 2020 were selected, including 100 patients with renal tumor, aged 57 to 72 years. There were 52 patients with prostate cancer, aged from 66 to 89 years. Forty-two patients with bladder tumor were aged 65–72 years. Some patients were with hypertension, hyperlipidemia, diabetes, chronic pulmonary obstructive disease (COPD), and other basic diseases. Patients were divided into observation group (*n* = 100) and control group (*n* = 100) according to the random number table method [12, 13].

### 2.2. Methods

Patients in the control group received routine preoperative and postoperative care and health guidance for corresponding diseases but did not do specific activity guidance and assistance. Patients in the observation group received comprehensive nursing intervention on the basis of the control group, including DVT and PE-wells risk assessment before admission (see Tables 1 and 2). Preoperative evaluation of comprehensive conditions (including blood sugar, blood lipid, blood pressure, and basic diseases.), 5 min Preoperative comprehensive evaluation (including blood glucose, blood lipid, blood pressure, and basic diseases): soak the patient's feet with warm water 5 min after operation (evaluate the anesthesia and the first activity of the patient's lower limbs), use traditional Chinese medicine for directional treatment of the lower limbs for 20 times, and guide the patient to actively exercise the contraction and relaxation of lower limb muscles, including hip, knee, and ankle flexed activity. The nursing operation is based on the principle of protecting the blood vessels of lower limbs, and the upper limbs are selected for venipuncture after surgery, avoiding nursing measures such as infusion in both lower limbs [14, 15].

### 2.3. Evaluation Indicators

The skin conditions (sclerosis and flushing), swelling, pain, deep vein thrombosis, and pulmonary embolism of lower limbs in 2 groups were observed within 5 to 15 days after surgery. The diagnosis of deep vein thrombosis of lower limbs is based on (1) the clinical manifestations of venous thrombosis in different parts, sudden swelling of one limb, accompanied by distension and superficial vein dilation. (2) Ultrasound Doppler detection results were used to determine thrombosis [16].

### 2.4. Statistical Methods

SPSS18.0 statistical software was used for data processing. Statistical data were compared using *X*^2^ test, and *P* < 0.05 was considered statistically significant [17].

## 3. Results

There was no significant difference in lower limb skin changes between the two groups (*P* > 0.05), and the incidence of lower limb swelling and pain in the observation group was significantly lower than that in the control group (*P* < 0.05). The incidence of DVT in observation group was significantly lower than that in control group (*P* < 0.05). In the control group, 3 patients had pulmonary embolism, and all died and were discharged. No pulmonary embolism occurred in the observation group. The difference was statistically significant [18–20] (see Tables 3 and 4).

## 4. Discussion

### 4.1. Analyze the Reasons

#### 4.1.1. Venous Thromboembolism

It has been included in the management category of the third batch of single diseases of National Health and Family Planning Commission. Venous thrombotic embolism (VTE) includes deep venous thrombotic embolism (DVT) and pulmona tythromboembolism. PTE (PTE) is a common disease in inpatients and often complicated with other diseases. It is an important cause of unexpected death in hospitals and has become a serious problem faced by hospital managers and clinical medical staff. According to the theory of venous thrombosis, it is generally believed that there are three inducing factors: slow blood flow, increased blood viscosity, and damage of venous intima.

#### 4.1.2. Surgical Factors

Urological laparoscopic and robot-assisted laparoscopic surgery patients need to inject gas into the abdominal cavity due to the special requirements of surgery. Intraabdominal pressure is maintained at 12∼15 mmHg (1 mmHg = 0.133 KPa) due to pneumoperitoneum during surgery. Higher pneumoperitoneum pressure can make the diaphragm move up, affect the return blood volume, reduce the contraction of the heart, make the whole blood flow stagnate, and increase the blood viscosity, resulting in the enhancement of coagulation function and eventually the formation of venous thrombosis especially in lower limb veins. Some of the 200 patients were older and complicated with hypertension, hyperlipidemia, diabetes, chronic pulmonary obstructive disease (COPD), and other basic diseases, while the hyperglycemia state can reduce the anterior microvascular resistance, increase the posterior microvascular resistance, and slow blood flow, microcirculation stagnation, and tissue hypoxia. Hyperlipidemia can increase blood viscosity and atherosclerosis so that blood is in a state of high coagulation. Therefore, the patient's blood is highly coagulable before surgery, which is high-risk factor for lower limb venous thrombosis after laparoscopic surgery.

#### 4.1.3. Postoperative Factors

Due to the blood loss and dehydration during the operation, the platelet blood concentration increased relatively and the adhesion increased, combined with the effect of postoperative hemostatic drugs, so that the blood is in a state of high coagulation. In addition, in order to prevent rebleeding after surgery, patients are required to rest in bed after surgery so that the limb activity is reduced, so that the venous blood flow slows down, blood stasis. Both increased the incidence of DVT in lower limbs.

#### 4.1.4. Malignancies

All 200 patients in this study were malignant tumors. Studies have shown that the hemorheology changes in patients with malignant tumor may be due to the direct activation of prothrombin by tumor cells through tissue factors or other procoagulant factors, thus initiating the exogenous coagulation pathway. Activation of platelets produces adhesion, aggregation, and release responses that predispose patients to lower extremity venous thrombosis.

#### 4.1.5. Surgical Body Position

During the operation, 200 patients were all in lithotomy position or side decubing position with high waist pad. The body was in excessive extension or excessive abduction of lower limbs for a long time, lower limbs were suspended for too long, and metal frame compression of lower limbs made venous return blocked and silted. At the same time, intraoperative position also aggravated the lower limb venous wall injury.

### 4.2. Robot-Assisted Laparoscopic Renal and Ureteral Surgery

(1) Robot-assisted laparoscopic renal (partial) resection: the earliest application of robot-assisted laparoscopic renal surgery was nephrectomy. For the first time, a 77-year-old woman was successfully performed renal resection under robot-assisted laparoscopy. The operation time was 245 min, and the blood loss was 100 ml. Robot-assisted laparoscopic partial nephrectomy can be performed more effectively in tumor resection and renal reconstruction with a safe thermal ischemia time of <30 min. Due to the unique advantages of robot-assisted laparoscopic surgery, kidney tumors can be completely and completely removed, while preserving the maximum normal kidney tissue. Wang et al. compared the differences between robot-assisted and purely laparoscopic partial nephrectomy, and the results showed that compared with purely laparoscopic, robot-assisted nephrectomy had shorter operation time, significantly shorter renal ischemia time and blood loss, and no significant difference in hospital stay. In terms of complications, laparoscopic alone was slightly higher than robot-assisted nephrectomy. At present, in order to maximize the protection of renal function, the time of renal artery occlusion should be shortened as far as possible. For renal tumors with small tumor diameter and obvious protrusion, partial nephrectomy can be performed without renal artery occlusion (called zero thermal ischemia). (2) The robot-assisted laparoscopic living donor nephrectomy: in living donor nephrectomy, laparoscopic techniques due to the small trauma, beautiful incision, postoperative recovery fast, and more open surgery have certain advantages, with the aid of Leonardo da Vinci robot-assisted surgery operation more stable; for free protection for renal blood vessels and ureter, reducing the separation for kidney damage is of great significance. (3) Robot-assisted laparoscopic pyeloplasty: with the assistance of da Vinci robot, the pruning of renal pelvis and the suturing of renal pelvis and ureter can be completed more quickly and more perfectly, shortening the operation time and improving the quality of surgery. Sung et al. first reported robot-assisted laparoscopic pyeloplasty in 1999. Olsen et al. reported 65 cases of robot-assisted pyeloplasty via retroperitoneal approach, with an average operative time of 143 min. They believed that the retroperitoneal approach was closer to the traditional pyeloplasty approach, with shorter operative time and the same therapeutic effect and complication rate as open surgery. Gettrnan et al. also compared it with traditional laparoscopic pyeloplasty, and the results showed that the operation time and suture time of the robot group were shorter than those of the traditional laparoscopic group.

### 4.3. Comprehensive Nursing Measures

**Figure 1:**
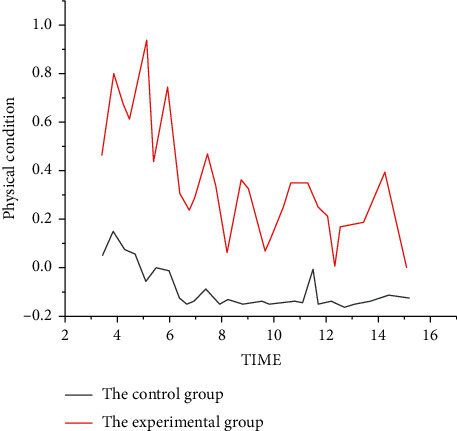
Comparison of the results between the two groups.

1. When patients in the observation group were admitted to hospital, in addition to preoperative routine examination, they focused on blood routine, coagulation series, and D-dimer examination. Preoperative risk assessment of DVT and PE-Wells (see Tables 1 and 2) and preoperative assessment of comprehensive conditions (including blood glucose, blood lipids, blood pressure, and underlying diseases) were carried out for patients with high-risk factors such as hypertension, hyperlipidemia, and diabetes. Preoperative treatment and monitoring of comorbidities should be strengthened and actively controlled.
2. Postoperative observation and preventive care for risk assessment and comprehensive evaluation of high-risk patients are strengthened. The patients' skin color, temperature, and sensorimotor condition of lower limbs were monitored. Ask the patient if the lower limbs are swollen and painful. After surgery, the upper limb vein was selected for intravenous puncture and the lower limb vein puncture was strictly prohibited without special circumstances.
3. On the day of surgery, targeted drug penetration therapy of traditional Chinese medicine was adopted, twice a day, 20 min each time. Routine treatment was performed for 3 days after surgery, and the treatment time was extended later according to the patient's condition. Directional drug penetration treatment of traditional Chinese medicine is through the original asymmetric intermediate frequency current of the electric field, promote the skin resistance drop, dilate small arteries and capillaries, improve local blood circulation, promote venous reflux, prevent blood stagnation, with thinning channels and collaterals, and regulate and improve circulation.
4. On the first day after surgery, the patients were given warm water soaking their feet and massage, once a day, 5 min each time, to promote the blood circulation of their lower limbs. It can effectively accelerate lower limb venous reflux, improve the internal environment of local tissue metabolism, increase endogenous fibrinolytic activity, and reduce the occurrence of lower limb deep vein thrombosis.
5. During the postoperative bed rest, a soft pillow can be placed at the ankle to avoid pillow pads under the knee, so as to raise the lower limbs, promote blood reflux, avoid gastrocnemius compression, and and guide the patient to do passive or active activities of lower limbs on the bed; the method is that the patient performs dorsiflexion, valgus, valgus, pronation, ankle pronation, knee joint, hip joint bending, hip joint pronation, and pronation spontaneously or with the help of the outside world. Exercise three to four times a day, 20 to 30 minutes each time. The early movement of lower limbs, especially knee joint extension and ankle active and passive movement, can increase the velocity of femoral vein blood flow. At the same time, it can prevent the aggregation of coagulation factors and the adhesion of vascular intima, which can effectively prevent the formation of DVT in lower limbs. Patients are assisted in stable condition to get out of bed early. In conclusion, comprehensive nursing intervention can effectively reduce the incidence of venous thromboembolism in patients with laparoscopic urology and robot-assisted laparoscopic surgery and effectively reduce the occurrence of venous thromboembolism and pulmonary embolism in lower limbs (Figure 1).

## 5. Conclusions

Objective is to explore the application effect of comprehensive nursing intervention in prevention of lower extremity deep vein thrombosis and pulmonary embolism in urological patients undergoing laparoscopic and robot-assisted laparoscopic surgery. From April 2019 to April 2020, 200 patients who received urological laparoscopic surgery and robot-assisted laparoscopic surgery were selected. According to the random number table method, they were divided into control group and observation group, 100 cases in control group and 100 cases in observation group. Patients in control group received routine nursing, while patients in observation group received comprehensive nursing intervention. The skin condition, swelling, pain, and occurrence of deep venous thrombosis and pulmonary embolism of lower extremities in 2 groups were observed. The experimental results showed that the lower limb swelling, lower limb pain, and lower limb deep vein thrombosis in the control group were significantly higher than those in the observation group, but all patients were cured and discharged after taking effective symptomatic treatment and nursing measures in time. In the control group, pulmonary embolism occurred in 3 patients, all of whom died. There was no significant difference except for skin changes of lower limbs (*P* > 0.05); other differences were statistically significant (*P* < 0.05). It proved that comprehensive nursing intervention can effectively prevent the formation of lower extremity deep vein thrombosis and pulmonary embolism in urological patients undergoing laparoscopic and robot-assisted laparoscopic surgery with high-risk factors.

## Figures and Tables

**Table 1:** Preoperative deep vein thrombosis (DVT) well clinical scores.

Clinical features	Points
Tumor active stage	1
Recently in bed and GT; major surgery is performed within 3 days or 4 weeks	1
The lower leg is swollen compared with the opposite side >3 cm	1
Ipsilateral superficial vein exposure (nonvaricose)	1
Swelling of the whole lower limb	1
Localized tenderness along a deep vein	1
There may be concave edema, and the lower limbs are heavier when measured with symptoms	1
Hemiplegia, paresis, recently performed lower limb plaster fixation	1
Symptoms over than DVT	–2
Total value	6

**Table 2:** Preoperative pulmonary embolism (PE) well clinical score.

Clinical features	Component
Previous history of PE or DVT	1.5
Heart rate > 100 times/min	1.5
Recent surgical procedures or braking	1
Clinical presentation of the DVT	1
A diagnosis of other diseases was less likely than for PE	1
Hemoptysis	1
Tumor	1
Total value	8

**Table 3:** Comparison of postoperative clinical manifestations of lower limbs of patients in the group.

Group	*n*	Lower limb skin condition				Harden		Blush	
		Lower limb swelling pain		Lower limb pain					
		Example	%	Example	%	Example	%	Example	%
Observation group	100	1	1	1	1	2	2	3	3
Control group	100	3	3	2	3	10	10	13	13

*X* ^2^	1.49					4.69		5.95	
*P*	>0.05					<0.05		<0.01	

**Table 4:** Comparison of postoperative DVT and PE between the two groups.

Group	*n*	Left lower extremity		Right lower extremity		Both lower extremities		Pulmonary embolism		Total amount	
		Example	%	Example	%	Example	%	Example	%	Example	%
Observation group	100	1	1	0	0.00	0	0.00	0	0.00	1	0.84
Control group	100	3	3	2	2	1	1	3	3	9	9

*X* ^2^	5.51										
*P*	<0.025										

## Data Availability

The data used to support the findings of this study are available from the corresponding author upon request.
